# Second brain: reviewing the gut microbiome’s role in lifestyle diseases

**DOI:** 10.5114/bta/195495

**Published:** 2025-03-31

**Authors:** Hindol Ray, Janatum Khatum, Srijan Haldar, Priyanka Bhowmik

**Affiliations:** 1 Department of Biological Sciences, Adamas University, Kolkata, West Bengal, India; 2 Department of Biotechnology, Swami Vivekananda University, Barrackpore, West Bengal, India

**Keywords:** gut microbiome, cancer, obesity, lifestyle diseases, diabetes, Alzheimer’s disease

## Abstract

The recent COVID-19 pandemic has highlighted another silent pandemic: lifestyle diseases. Conditions, such as cardiovascular diseases, anxiety, and type 2 diabetes (T2D), are increasingly becoming public health threats, affecting even younger populations worldwide. In recent years, extensive research has uncovered the pivotal role of the human gut microbiome in various aspects of human physiology, including metabolism, cellular homeostasis, immune defense, and disease development. The gut microbiome, often referred to as the “second brain,” is now recognized as a key player in health and disease. Lifestyle factors such as diet, mental health, stress, exercise, and others significantly influence the composition of the gut microbiome. Imbalances in this composition, termed “dysbiosis,” have been linked to a wide range of diseases, including cancer, cardiovascular diseases, obesity, T2D, asthma, and neurological disorders like Alzheimer’s and Parkinson’s disease. These findings underscore the profound influence of gut microbiome health on overall well-being. A working understanding of the gut microbiome’s composition and its impact on disease processes is crucial for the advancement of personalized or precision medicine. This review article aims to explore recent advancements in the field, shedding light on how the gut microbiome contributes to the development and prognosis of lifestyle diseases.

## Introduction

The human gastrointestinal tract harbors trillions of resident microbes, collectively known as the gut microbiome. Advances in next-generation sequencing, highthroughput sequencing platforms, and mass spectrometry have provided significant insights into the gut microbiota, its diversity, and the myriad microbial metabolites that play essential roles in human biology (Durack and Lynch, 2019). The gut microbiota is highly sensitive to its environment; it synthesizes a wide array of biochemicals, from small diffusible molecules to large, complex biomolecules such as macrolides and polyketides (Donia and Fischbach, 2015). This biochemical arsenal sustains the intricate interactome of the gut microbiota, governed by a combination of host genetics and environmental factors. Diet (Kamiya et al., 2018), antimicrobial agents, stress, sleep cycles, and physical activity levels influence the diversity and variation of the gut microbiome in complex ways (Wheeler et al., 2016; Ticinesi et al., 2017; Bu and Wang, 2018).

Lifestyle diseases, linked to daily activities, are increasingly common due to sedentary behaviors, fast-food diets, and excessive antibiotic usage. Rapid urbanization has further exacerbated the prevalence of these diseases, which are now the leading causes of death worldwide.

**Figure 1:**
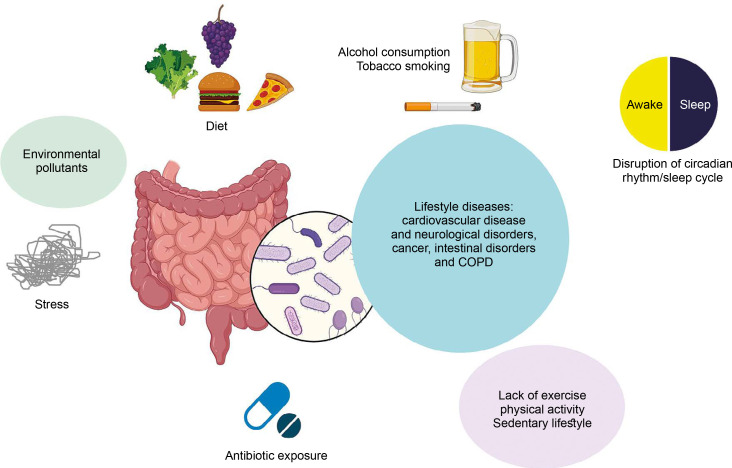
Close relationship between lifestyle factors, gut microbiome, and various diseases

These observations point to a close relationship between lifestyle factors, the gut microbiome, and the onset and progression of diseases such as cardiovascular diseases, chronic obstructive pulmonary disease (COPD), and metabolic disorders (Zhang et al., 2015) (Figure 1).

This review summarizes the current understanding of how the gut microbiome influences health and disease, examining the link between gut microbiome dysbiosis and the development of lifestyle-related diseases. It also explores how environmental cues impact disease processes through their effects on gut microbiome composition and activity.

## Sources and selection criteria

To conduct this review, we performed a comprehensive literature search using databases such as PubMed and Google Scholar. The following search terms were used in various combinations: gut microbiome, gut microbiota, gut-brain axis, gut-liver axis, short chain fatty acids (SCFAs), ulcerative colitis (UC), Crohn’s disease (CD), inflammation, bacteriotherapy, fecal microbiota transplant, cancer, Parkinson’s disease (PD), obesity, diet, lifestyle, mucosal immunity, *Bifidobacterium*, *Lactobacillus*, antibiotics, germ-free mice, exercise, cardiovascular disease, epigenetic regulation, pro-inflammatory cytokines, dysbiosis, inflammatory bowel disease (IBD), circadian rhythm, sleep, smoking, alcohol abuse, type 2 diabetes mellitus (T2D), Alzheimer’s disease (AD), asthma, and COPD. No restrictions were placed on publication dates. Additionally, reference sections of the articles were reviewed to identify and gather related information. The study included various types of articles, such as meta-analyses, cross-sectional, casecontrol, observational, and population-based studies, as well as open-label, placebo-controlled, and randomizedcontrolled trials.

## Association of diseases with the lifestyle

Many diseases share behavioral risk factors that contribute to their progression. Key lifestyle practices that negatively impact human health are discussed in the following section.

### Tobacco smoking

Tobacco smoking is strongly associated with the progression of cardiovascular diseases (CVD) and contributes significantly to related mortality and morbidity. It is a leading cause of coronary artery disease and cerebrovascular disease (Rigotti and Clair, 2013). Smoking is also a well-established risk factor for cancer development. Furthermore, a multivariate linear regression study demonstrated the adverse effects of smoking on body mass index and obesity (Ellulu, 2018). Interactions between genetic loci and smoking have been shown to influence obesity through various biological pathways (Lee et al., 2022). Cigarette smoke contains thousands of harmful chemicals, including free radicals and tars, which induce inflammatory responses and increase oxidative and nitrosative stress. These markers of inflammation and stress responses may also increase susceptibility to depression in smokers (Berk et al., 2013).

### Diet

Dietary habits have a profound influence on the development of various diseases, including cancer. For instance, poor diet and nutrition have been linked to ovarian cancer in female patients (El-Sherif et al., 2021). Animal studies demonstrate that feeding mice a high-fat diet triggers the expression of sodium-glucose cotransporter-1, which impairs glucose homeostasis in the small intestine and leads to increased endogenous glucose production by the liver, ultimately contributing to diabetes. Conversely, restricted diets such as the ketogenic diet have shown promising results, particularly in managing neurological disorders such as AD and PD in small cohort studies (Stafstrom and Rho, 2012).

### Sleep

Abnormal sleep patterns or sleep deprivation are linked to the pathophysiology of psychiatric disorders, including depression. Studies indicate that acute sleep deprivation upregulates pro-inflammatory cytokines such as TNF-α and IL-6, impairing immune function. These biological markers are closely associated with depressive disorders (Shearer et al., 2001).

### Vitamin D

Lower levels of vitamin D, particularly 25-hydroxyvitamin D, are prevalent in Western populations and are associated with an increased risk of cancer, depression, and osteoporosis. Vitamin D plays a role in reducing inflammatory markers such as TNF-α and IL-6, which are implicated in many disorders (Beilfuss et al., 2012).

### Stress and trauma

Psychological stressors induce systemic and central nervous system (CNS) levels of pro-inflammatory cytokines, including IL-1 and IL-6. Additionally, studies report stress-induced increases in IFN-γ and Th-1 dominance, which are correlated with anxiety and depression.

### Exercise

Physical activity has been shown to provide protective effects against various types of tumors, including breast and reproductive tract tumors (Shephard and Freedson, 1996).

### Alcohol consumption

Alcohol consumption is linked to an increased risk of several cancers, including cancers of the oral cavity, esophagus, pharynx, larynx, liver, colorectum, and breast (Choi, Myung and Lee, 2018).

### Disruption of the circadian rhythm

The circadian rhythm is a 24-h cycle that regulates metabolic, synthetic, and signal transduction pathways. Disruption of this rhythm has been linked to various types of cancer, although the mechanisms remain elusive (Altman, 2016).

### Use of antibiotics

Abrupt and excessive use of antibiotics not only promotes widespread antibiotic resistance but also increases the risk of cancers. A large-scale cohort study demonstrated an association between antibiotic use and an elevated risk of cancer (Kilkkinen et al., 2008).

### Environmental pollution

Environmental pollution is a well-established risk factor for non-communicable diseases, including cancer. For instance, the incomplete combustion of fossil fuels is linked to increased lung cancer incidence. Outdoor air pollution has also been associated with poorer survival rates among cancer patients (Cohen and Pope, 1995).

Environmental factors and individual lifestyle choices, such as diet, exercise, alcohol consumption, and sleep patterns, play critical roles in disease development and prognosis in humans. However, understanding the interplay between these environmental factors and an individual’s genetic makeup remains challenging. Vertebrates interact with their external environment through various interfaces, including mucosal layers in the airways, skin, and gastrointestinal tract. Among these, the gut is the largest immune organ and serves as a critical interface with the external environment, shaping immune regulation in the host.

The gut’s commensal microbiota, or the “gut microbiome,” is highly sensitive to environmental stimuli, including diet, delivery method at birth, rearing practices, number of siblings, and other lifestyle factors (Shi et al., 2017). These stimuli influence host health and disease.

**Figure 2:**
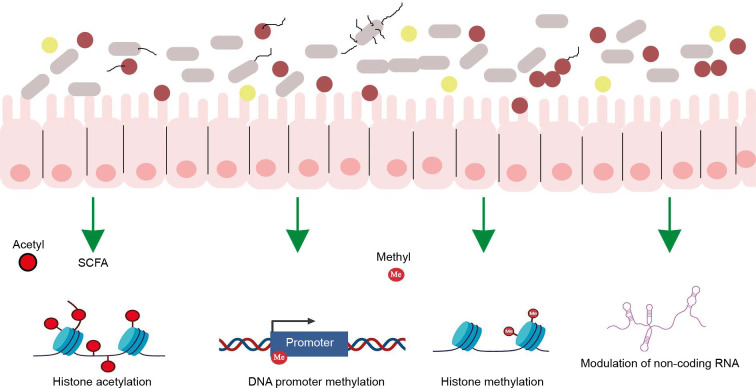
Interaction of gut microbiota with human epigenome. The human gut microbiota synthesizes various epigenetic substrates and/or modulators of chromatin-modifying enzymes [Adapted from Woo and Alenghat, 2022]

The gut microbiome employs various mechanisms to regulate host interaction with the environment, with epigenetic regulation being one of the most critical. The gut microbiome calibrates host metabolism and transcription through epigenetic regulation via three main mechanisms: a) microbial metabolism influencing DNA or histone modification, b) regulation of epigenetic-modifying enzyme expression and activity, and c) activation of host cell epigenetic pathways (Figure 2).

### Microbial metabolism influencing DNA or histone modification

The gut microbiota synthesizes numerous molecules that serve as substrates or cofactors for epigenetic regulation in the host. For example, the gut microbiota synthesizes folate, which donates methyl groups for DNA or histone modification (Rossi, Amaretti and Raimondi, 2011).

### Regulation of epigenetic-modifying enzyme expression

Gut bacteria produce SCFAs through the fermentation of nondigestible complex carbohydrates and fibers. These SCFAs alter the host’s gene expression profile by inhibiting histone deacetylase activity, which causes chromatin changes that are involved in enhanced gene expression (Krautkramer et al., 2016).

### Activation of host cell epigenetic pathways

The intestinal microbiota regulates DNA methylation and demethylation, critical processes for controlling gene expression (Takahashi et al., 2009). Additionally, the gut microbiome influences histone modifications, which influence chromatin conformation and subsequent gene expression. The microbiome also modulates noncoding RNAs (nc RNAs), which play a role in gene expression regulation (Liang et al., 2015; Woo and Alenghat, 2022).

Furthermore, the gut microbiome is pivotal in forming mucosal immunity, which is essential for maintaining host homeostasis and defense. SCFAs synthesized by gut microbes exert anti-inflammatory effects by inhibiting histone deacetylase activity in regulatory T cells (Tregs) via G protein-coupled receptors (GPCRs). The gut microbiome also influences the host by synthesizing a wide array of metabolites.

## Human gut microbiome constituents

The human microbiome consists of 10–100 trillion symbiotic microorganisms, including eubacteria, archaea, viruses, eukaryotic cells, bacteriophages, fungi, and their associated genomes, genes, and gene products.

The number and composition of gut microbiota vary throughout the gastrointestinal tract. The stomach and proximal duodenum host relatively few bacteria (approximately 10^1^ bacteria per gram of content), whereas the colon harbors a significantly higher concentration (10^12^ bacteria per gram). The gut microbiota is predominantly composed of Gram-negative anaerobes from the phylum *Bacteroidetes* (48%) and Gram-positive *Firmicutes* (51%). The remaining 1% is highly variable, including *Cyanobacteria*, *Fusobacteria*, *Proteobacteria*, *Spirochaetes*, *Verrucomicrobia*, various viruses, bacteriophages, fungi, and protozoa (Manrique *et al.*, 2016; Dieterich, Schink and Zopf, 2018).

## Lifestyle diseases affected by the gut microbiome

The gut microflora interferes with every aspect of health. Lifestyle diseases are noncommunicable diseases and are primarily based on the day-to-day habits of people. Lifestyle diseases include CVD and neurological disorders, cancers, intestinal disorders, and COPD. The lifestyle diseases influenced by gut microbiome can be stratified into two groups: intestinal disorders and extraintestinal disorders. The following section discusses the interrelationship of external lifestyle factors, gut microbiome, and the development and prognosis of various diseases. Table 1 tries to demonstrate this interrelationship.

**Table 1:** Interrelationship of lifestyle risk factors, gut microbiome modulation, their role in the genesis, and prognosis of diseases

Risk factor	Associated disease	Potential microbe-related mechanism
Obesity	Colorectal cancer	Increased LPS level in the gut, epigenetic changes in colonic epithelial cells, and increased production of bile acids that damage DNA
	Hepatocellular carcinoma	Increased production of deoxycholic acid that causes DNA damage by generating ROS
Diet	Colorectal cancer	Changes in the acetylation pattern e.g. lysine 27 on histone H3 (H3K27ac) and altered monomethylation of lysine 4 on histone H3 (H3K4me1)
	Celiac disease	Aberrant immune reaction
	IBD	Increase in sulfate-reducing bacteria, ↑ production of H2S, and mucosal toxicity
	Alzheimer’s disease	Diet rich in polyphenols decreases inflammation, plant-based diet increases the production of neuroprotective SCFAs
	Liver cancer	Malnourished diet can cause oxidative stress, high cholesterol diet results in ↑ N, N, N-trimethyl-5-aminovaleric acid (TMAVA) that ↑ inflammation
	Gastric cancer	↑ Inflammation, ↑ mutation ROS-mediated DNA damage, angiogenesis
	Prostate cancer	↑ in IGF1 ↑ cancer cell proliferation
	Esophagus cancer	Carcinogenic H2S production, carcinogenesis by TLR pathway
	Type 2 diabetes	Expansion of inflammatory bacterial population
	Cardiovascular disease	Vegetarian diet enhances the production of beneficial SCFAs while a Western diet causes the production of TMAO; ↑ pro-inflammatory cytokines, and cholesterol accumulation in the arterial wall
	Asthma	Breast-fed children ↑ microbial diversity and high protection, formula-fed children ↓ low microbial diversity and low protection
Sleep, circadian rythm	Neurological disorders	Shift in gut microbial community
	Type 2 Diabetes	Loss of rhythmicity in gut microbiota and loss of metabolic control
	Colorectal cancer	Accumulation of MDSCs triggering lung metastasis of colorectal cancer
Smoking	IBD	Increases inflammation by changing the microbial composition
	COPD	↓ Level of SCFAs, ↓ histone deacetylase 2 causing inflammation
	Colorectal cancer	↑ Inflammatory IL-17 and TNF-α pathways
Antibiotic exposure	IBD	Use of antibiotics in childhood associated with IBD development
Alcohol consumption	Pancreatic cancer	↑ Acetaldehyde, ↑ superoxide, ↑ inflammation
Physical Exercise	Cardiovascular disease	↓ Inflammation
	Alzheimer’s disease	↑ SCFAs

## Intestinal disorders

### Inflammatory bowel disease

IBD is characterized by uncontrolled intestinal inflammation, with only 25% of cases explained by genetic predisposition. Environmental and lifestyle factors such as diet, stress, and smoking significantly influence IBD development. Recent studies highlight alterations in gut microbiota composition and function in IBD. Depending on the inflammation site, IBD is classified into CD and UC. The gut microbiome influences these diseases in the following manners: a) microbial dysbiosis, b) metabolic effects, c) effect on immunity, d) barrier disruption and e) enteric nervous system dysbiosis.

#### Microbial dysbiosis

IBD leads to gut microbiome dysregulation, reducing bacterial community richness and promoting the proliferation of facultative anaerobic *Enterobacteriaceae* (Lewis et al., 2015).

#### Metabolic effects

Microbiota-derived metabolites, including SCFAs, bile acid metabolites, and tryptophan metabolites, play critical roles in IBD (Lavelle and Sokol, 2020). The abundance of the pathogenic genera *Treponema* and *Fusobacterium* can be correlated with the production of hydrogen sulfide, an immunomodulator that regulates the host inflammatory response (Segata et al., 2012). Additionally, IBD-associated microbiota sequesters magnesium, whose deficiency is a known side effect of IBD.

#### Effect on immunity

Gut microbes regulate the balance of Th17 and Treg cells, crucial for maintaining intestinal homeostasis (Khan et al., 2019). A study using a mouse model showed that microbial production of sphingolipids controls the inflammatory activity of natural killer (NK) cells, which is positively correlated with decreased severity of UC (An et al., 2014).

#### Barrier disruption

IBD disrupts the intestinal physical barrier, exposing host cells to pathogenic bacteria.

#### Effect on enteric nervous system

Gut microbes synthesize neuroactive molecules such as γ-aminobutyric acid (GABA), tryptamine, indole-3-propionic acid, serotonin, and SCFAs. These molecules influence the enteric nervous system and help maintain intestinal homeostasis.

Dietary and lifestyle changes play a significant role in influencing the gut microbiome, often predisposing individuals to IBD. The Western diet, characterized by high meat consumption, disrupts the gut microbial community, shifting it toward IBD-associated types (Lee and Chang, 2021). A diet high in saturated fat promotes the proliferation of sulfate-reducing bacteria, leading to increased production of mucosally toxic hydrogen sulfide, a contributor to ulcerative colitis. Similarly, high-fat diets alter gut microbiota composition, increasing the abundance of pathobionts and plasma endotoxin levels. Additionally, obesity causes a reduction in anti-inflammatory gut microbiota and metabolites, which might trigger innate immunity to secrete proinflammatory cytokines through pattern recognition receptor-mediated pathways (Kim, Oh and Yoo, 2023). Food additives such as carrageenan exacerbate this issue by serving as a sulfur source for bacteria like *Bilophila wadsworthia to* produce H_2_S, which results in inflammation. Smoking further compounds the problem, particularly in CD, as it shifts the gut microbiome toward a pro-inflammatory landscape. Metagenomic studies have revealed a reduction in beneficial bacteria such as *Faecalibacterium prausnitzii*, which produces anti-inflammatory proteins (Shapiro et al., 2022). Moreover, early-life exposure to antibiotics has been linked to an increased risk of IBD development (Kronman et al., 2012).

### Celiac disease

Celiac disease is an intestinal inflammatory disorder caused by an aberrant immune response to dietary wheat gluten proteins in genetically predisposed individuals. The disease is associated with a decreased abundance of *Bifidobacterium* and an increased presence of *Staphylococcus* and *Bacteroides fragilis*, which may contribute to disease progression. An Italian study reported a strong correlation between celiac disease, intestinal infections, and antibiotic use. In this condition, the mucosal layer fails to protect the host from invading pathogens such as *Bacteroides* and *E. coli* (De Palma et al., 2010).

### Colorectal cancer

Colorectal cancer is a leading cause of mortality worldwide, strongly associated with diet and lifestyle factors such as red and processed meat consumption, alcohol intake, obesity, and smoking. While the exact mechanisms linking these factors to cancer remain unclear, evidence suggests that gut microbiota mediates this relationship. High-fat diet-induced obesity, for instance, leads to gut microbiota-dependent remodeling of acetylation patterns in cis-regulatory regions, altering gene expression profiles. Obesity-related Gram-positive gut bacteria stimulate the production of secondary bile acids, such as deoxycholic acid, which cause DNA damage through reactive oxygen species, promoting cancer development (Song and Chan, 2019). Smoking is another contributor to gut microbiome dysbiosis, marked by an increase in *Eggerthella lenta* and *Staphylococcus capitis* and a decrease in *Parabacteroides distasonis* and *Lactobacillus* species. This dysbiosis correlates with elevated levels of bile acids like taurodeoxycholic acid (TDCA) in the colon, leading to barrier disruption, and the upregulation of inflammatory pathways, including IL-17 and TNF-α, in colonic epithelial cells (Shapiro *et al.*, 2022). Recent research has uncovered a link between the gut microbiome and the circadian rhythm in myeloidderived suppressor cells (MDSCs), which facilitate lung metastasis of colorectal cancer. Both the biological clock of these cells and gut microbiota-derived metabolites, such as taurocholic acid, promote MDSC accumulation by epigenetically enhancing glycolysis (Liu et al., 2024).

## Extra-intestinal disorders

### Cancer

Cancer claims over 7 million lives annually worldwide, with more than 30% of cases attributable to lifestyle choices. Factors such as smoking, alcohol consumption, obesity, and diet are significant contributors. Smoking alone accounts for 20–30% of cancer cases (Katzke, Kaaks and Kühn, 2015). The diet also plays a critical role, with studies on gynecological cancers demonstrating a protective effect from consuming vegetables and fruits. Conversely, obesity or a high body mass index increases the risk of mortality from ovarian or cervical cancer. Other lifestyle factors, such as alcohol consumption, lack of physical activity, and hormonal contraceptive use, are also associated with cancer risk (Rieck and Fiander, 2006).

The complex cross-talk between the microbiome and the host regulates both the genesis and prognosis of cancer. Microbe-derived SCAFs have demonstrated anticancer effects, and bacterial lipopolysaccharides are known to activate T cell-mediated immune responses against cancer cells. However, gut microbiome dysbiosis can also contribute to cancer development. Gastrointestinal dysbiosis has been linked to both local and distant tumors. Pathogenic gut bacteria, such as *Shigella*, can interfere with host DNA damage response and repair pathways, increasing the likelihood of mutations that may lead to tumor formation. Furthermore, certain bacteria can disrupt host proliferative pathways. For instance, *Fusobacterium nucleatum* effector adhesin A (FadA), *Bacteroides fragilis* metalloproteinase toxin (MP toxin), and *Helicobacter pylori* Cag A protein have been implicated in altering cellular proliferation, ultimately contributing to cancer development (Vivarelli et al., 2019).

**Figure 3:**
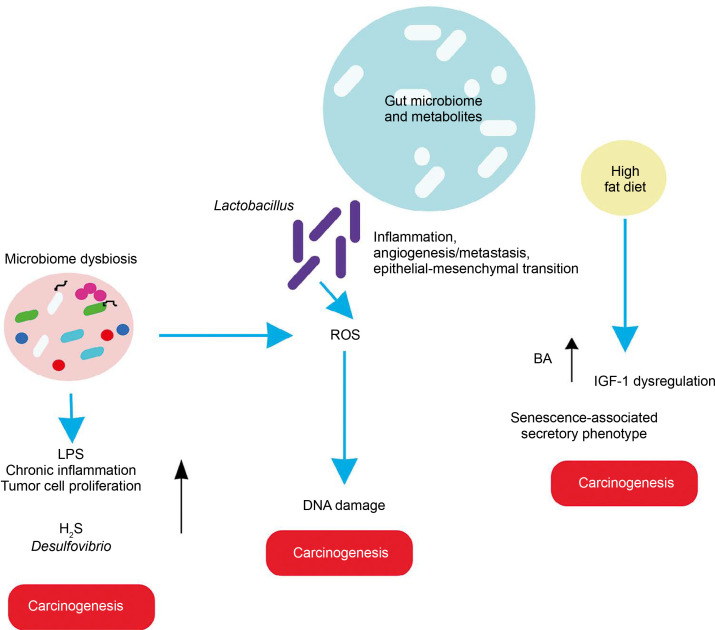
Relation between altered gut microbiome and different cancers

In light of these findings, the role of gut microbiota in various cancers is elaborated below, with basic mechanisms depicted (Figure 3).

### Liver cancer

The gut and liver are interconnected via the portal vein, forming the entero-hepatic circulation or “gut-liver axis,” which is crucial for liver homeostasis and the prognosis of liver diseases. A study revealed that a high-cholesterol diet leads to the accumulation of N,N,N-trimethyl-5-aminovaleric acid (TMAVA), which induces liver inflammation and may be linked to hepatic carcinoma (Zhang et al., 2021). Additionally, dietary shifts or malnourishment can cause gut microbiome dysbiosis, leading to aberrant ROS generation and oxidative stress in the liver.

### Esophagus cancer

A high-fat diet promotes esophageal cancer by inducing inflammatory responses. For instance, *Fusobacterium nucleatum* may contribute to aggressive tumor development by activating chemokines like CCL20 (Baba et al., 2017). High-fat diets promote the production of carcinogenic H_2_S by *Desulfovibrio* spp. and *Clostridium lavalense*, which promote cancer by upregulating HSP90. Moreover, these diets activate IL-8 via Toll-like receptor (TLR) signaling, further driving cancer progression. Chronic inflammation caused by gut microbial shifts induced by high-fat diets leads to metaplasia and dysplasia (Münch et al., 2019).

### Gastric cancer

High-fat diets encourage the growth of *Lactobacillus* (LAB), which produces lactic acid that acidifies the gastric mucosal surface. This lactic acid serves as a fuel source for cancer cells and promotes inflammation, angiogenesis, metastasis, and epithelial–mesenchymal transition. LAB also generates ROS, leading to DNA damage. Additionally, LAB converts nitrates to nitrites, which induce mutations, reduce apoptosis, promote angiogenesis, and enhance proto-oncogene expression. These mechanisms also create immune tolerance, allowing colonization and proliferation of carcinogenic pathogens such as *Veillonella*, *Prevotella*, *Fusobacterium*, and *Leptotrichia*, thereby promoting tumorigenesis. High-fat diets further support the overgrowth of endotoxin-producing *Enterobacteriaceae*, triggering inflammatory pathways (Tong et al., 2021).

### Breast cancer

Studies indicate that a high-fat diet influences gut microbiota, contributing to poor prognosis and advanced clinicopathological features in breast cancer patients (Chen et al., 2024).

### Prostate cancer

In a prostate-specific *Pten* knockout mouse model, high-fat diets were shown to cause gut microbial dysbiosis. This dysbiosis regulated insulin-like growth factor-1 (IGF-1) through SCFAs, promoting the proliferation of prostate cancer cells (Matsushita et al., 2021).

### Pancreatic cancer

Heavy alcohol consumption alters bile acid levels, leading to changes in gut microbiota composition. This disruption increases intestinal permeability and triggers systemic inflammatory reactions. High dietary fat consumption enhances the production of the secondary bile acid deoxycholic acid by 7α-dehydroxylating gut bacteria, which accelerates the senescence-associated secretory phenotype and promotes genomic instability and cancer. Obesity is correlated with a decrease in overall microbial diversity of the gut microbiome, which causes changes in metabolic pathways and subsequently contributes to the development of cancer (Li *et al.*, 2020). Acetaldehyde, an alcohol byproduct, generates superoxide and toxic metabolites that induce inflammatory reactions, potentially leading to pancreatic cancer.

### Metabolic disorders

#### Obesity

Obesity is a global public health crisis, affecting 603.7 million adults worldwide, and is associated with various comorbidities such as CVD, T2D, and cancer (Haslam, Sattar and Lean, 2006; Lee, Sears and Maruthur, 2020). While obesity is influenced by genetic, nutritional, personal, and environmental factors, the imbalance of gut microbiota plays a crucial role in its development (Zhao, 2013; Gupta, Osadchiy and Mayer, 2020). Obesity is often associated with altered taxonomical composition in gut microflora, characterized by the expansion of opportunistic pathogens. Obese individuals generally have higher levels of *Firmicutes* and *Bacteroides* compared to lean individuals, although studies have reported contradictory findings regarding the relative abundance of *Bacteroides* (Kasai et al., 2015; Oduaran et al., 2020). Thirteen genera, including *Acidaminococcus*, *Anaerococcus*, *Catenibacterium*, *Dialister*, *Dorea*, *Escherichia-Shigella*, *Eubacterium*, *Fusobacterium*, *Megasphaera*, *Prevotella*, *Roseburia*, *Streptococcus*, and *Sutterella*, are more abundant in obese individuals, whereas *Bifidobacterium* and *Eggerthella* are significantly less abundant. Yeast predominates in the gut of obese individuals, while *Trichosporon* species are more abundant in nonobese populations.

The Firmicutes-to-Bacteroidetes ratio (F/B ratio) is considered a biomarker for obesity predisposition (Tseng and Wu, 2019). A metagenomic study in a cohort of Chinese individuals identified an obesity-associated bacterium, *Bacteroides thetaiotaomicron*, which alleviates diet-induced body weight gain (Chen et al., 2023).

#### Type 2 diabetes

T2D is a prevalent metabolic disorder characterized by imbalances in blood glucose levels, lipid profiles, and high blood pressure (BP). It results from insulin nonresponsiveness and insufficient insulin secretion due to reduced β-cell function. Numerous studies have linked intestinal dysbiosis to the onset and progression of T2D. Changes in dietary patterns alter the Firmicutes-to-Bacteroides ratio within the gut microbiome, with T2D patients often exhibiting an increase in pro-inflammatory bacteria such as *E. coli* and a reduction in anti-inflammatory bacteria like *Faecalibacterium prausnitzii*. This imbalance is associated with chronic low-grade inflammation, or “metainflammation,” in T2D.

Gut microbiota contributes to T2D by degrading undigestible carbohydrates into SCFAs. These SCAFs, along with bacterial LPS, activate inflammatory networks in the gut. Although no direct link between inflammation and T2D has been established, inflammation strongly correlates with insulin unresponsiveness, which, in turn, leads to T2D. Pro-inflammatory cytokines, such as IL-1β, further exacerbate the condition by damaging insulinproducing β-cells (Kwon and Pessin, 2013; Sikalidis and Maykish, 2020). Oxidative stress is also linked to glycation phenomena and the onset of diabetes (Tsalamandris et al., 2019). A recent study on mice fed a high-fat diet, with or without a lipoic acid supplement – a known antioxidant – showed that the supplemented group had a better B/F ratio, reduced oxidative stress, and, consequently, a lower risk of developing associated chronic disorders such as T2D (Larsen et al., 2010).

Diabetes medication, particularly metformin, has been shown to positively influence gut microflora. A metagenomic study involving 345 volunteers found that diabetes patients exhibit a decrease in butyrate-producing bacteria and an increase in opportunistic pathogens compared to healthy individuals. Treatment with metformin causes rapid alterations in gut microbiome composition, as evidenced by a placebo-controlled study (Qin et al., 2012; Wu et al., 2017). Metformin also exerts its effects through circadian rhythm intervention by activating the AMP-activated protein kinase (AMPK) pathway. The circadian rhythm, which aligns daily food intake and digestion with the day-night cycle, is crucial for metabolic health. Dysregulation of this rhythm is associated with T2D. A seminal study identified a disruption of rhythmicity in gut microbiota in T2D patients, highlighting a risk pattern of anti-rhythmic taxa that contribute to the disease. This loss of rhythmicity in the gut microbiota correlates with impaired metabolic control in T2D patients (Reitmeier et al., 2020).

### Cardiovascular diseases

CVD, encompassing hypertension, atherosclerosis, cardiomyopathy, and heart failure, are leading cause of mortality and morbidity worldwide. Emerging evidence underscores the pivotal role of gut microbiota in regulating these diseases, while CVD itself impacts gut microbial composition. For instance, hypertensive patients exhibit significantly different gut microbial clustering and reduced diversity, with lower α-diversity (Ahmad et al., 2019) and a higher abundance of *Prevotella* species. Similarly, metagenomic studies have shown that individuals with atherosclerotic CVD have elevated levels of *Streptococcus* and *Enterobacteriaceae* species compared to healthy individuals (Emoto et al., 2017). Heart failure patients show an increased prevalence of pathogenic bacteria such as *Campylobacter*, *Shigella*, *Salmonella*, and *Yersinia enterocolitica*. The severity of heart failure has also been associated with the presence of *Candida*, *Campylobacter*, and *Shigella* (Pasini et al., 2016).

Pro-inflammatory microbiome alterations impair cholesterol and lipid metabolism, reducing levels of protective metabolites like SCFAs. SCFAs help prevent cardiovascular damage by reducing systolic BP, lowering serum cholesterol, and maintaining an anti-inflammatory microenvironment (Xu et al., 2020).

Lifestyle factors significantly influence CVD risk and also alter gut microbiota composition. For example, adherence to a vegetarian, high-fiber, low-fat diet has been shown to increase SCFA production compared to diets rich in animal protein, low in fiber, and high in fat (De Filippo et al., 2010). In contrast, microbial metabolism of Western diet components like red meat, egg yolks, and animal products produces trimethylamine N-oxide (TMAO), a metabolite associated with higher CVD risk and mortality postheart failure. TMAO promotes cholesterol accumulation in arterial walls, reduces cholesterol clearance from peripheral endothelial cells, and enhances vascular inflammation through proinflammatory cytokine expression (Janeiro *et al.*, 2018). Phenylalanine, found in dietary proteins, is metabolized into phenylacetylglutamate, which interacts with β-adrenergic receptors, mediating platelet aggregation and increasing the risk of heart attack, stroke, and death (Nemet et al., 2020; Witkowski, Weeks and Hazen, 2020). Physical activity also protects against CVD. Studies on rugby athletes revealed lower inflammation, increased microbial diversity, and enrichment of beneficial taxa such as *Ruminococcaceae*, *Succinivibrio*, and *Akkermansia*. Studies showed the correlation of a major shift in the day and night pattern of microbiota with the rhythmicity of BP. Another study demonstrated the close association of sleep fragmentation and elevated bacterial population associated with changes in mean arterial pressure, and abundance of certain fecal bacterial metabolites and BP. All these lead to links to disturbed sleep and cardiovascular pathology (Maki et al., 2020). Studies also demonstrated that circadian regulation of the gut microbiome is important in cardiac repair postmyocardial infarction (Gumz et al., 2023).

### Respiratory diseases

#### Chronic obstructive pulmonary disease

COPD is a heterogeneous disease characterized by chronic bronchitis, airway remodeling, and emphysema, often accompanied by systemic comorbidities such as CVD, colitis, osteoporosis, and even cancer (Caramori et al., 2019). The gut-lung axis, which comprises the common mucosal immune system of the gut and lungs, regulates inflammation in COPD. The gut microbiome significantly influences host responses to respiratory illnesses (Trompette et al., 2018; Vaughan et al., 2019). COPD patients exhibit a distinct gut microbiome composition compared to healthy individuals, with altered diversity identified through 16S rRNA sequencing. Genera such as *Streptococcus*, *Rothia*, *Romboutsia*, *Intestinibacter*, and *Escherichia* are increased in COPD patients, while *Bacteroides*, *Roseburia*, *Lachnospira*, and several unnamed *Ruminococcaceae* (Bowerman et al., 2020) genera are reduced (Bowerman et al., 2020). Notably, elevated levels of gut microbiota-derived TMAO have been observed in the serum of severe COPD patients, linking this metabolite to disease progression (Ottiger et al., 2018).

Smoking, a primary risk factor for COPD, disrupts gut microbiota, reducing overall diversity and increasing the relative abundance of pro-inflammatory bacteria like *Prevotella*. Smoking also diminishes levels of SCFAs, which possess anti-inflammatory properties (Li et al., 2021). Additionally, COPD patients have reduced levels of histone deacetylase 2 (HDAC2) compared to the non-smoker population, which can be correlated with inflammation in COPD.

Malnourishment is an overlooked factor in COPD. A lack of dietary fiber causes dysbiosis in the gut microbiota and reduces the production of anti-inflammatory SCFAs.

#### Asthma

Asthma, a chronic respiratory condition, affects over 300 million people worldwide. An imbalance between symbiotic and pathogenic gut microbiota has been correlated with the development of asthma. Gut microbes produce metabolites with both pro-inflammatory and anti-inflammatory effects, such as biogenic amines (e.g., histamines) and oxylipins (e.g., 12,13--diHOME). Asthma patients exhibit a higher abundance of histamine-producing bacteria compared to healthy individuals, with these levels correlating with disease severity (Levan et al., 2019; Barcik et al., 2020). Gut bacteria also play a critical role in modulating T helper and effector cell populations, which are crucial (very important) in the manifestation of allergic asthma (Di Gangi et al., 2020). Low gut microbial diversity is closely associated with asthma, as evidenced by studies showing that breastfed children, who typically have greater gut microbial diversity, have a lower risk of asthma compared to formula-fed children (Oddy, 2017).

### Neurological disorders

#### Alzheimer’s disease

AD is a progressive neurodegenerative disorder characterized by memory loss, inability to perform daily tasks, and continuously diminishing cognitive functions (Alzheimer’s Association, Thies and Bleiler, 2013; Askarova et al., 2020). In the early 90s, the amyloid cascade hypothesis emphasized that the AD pathophysiology is attributed to the deposition of amyloid particles in the brain. However, the amyloid cascade hypothesis is failing with more and more documented studies that show no correlation between amyloid particle deposition and clinical manifestation of the disease.

**Figure 4:**
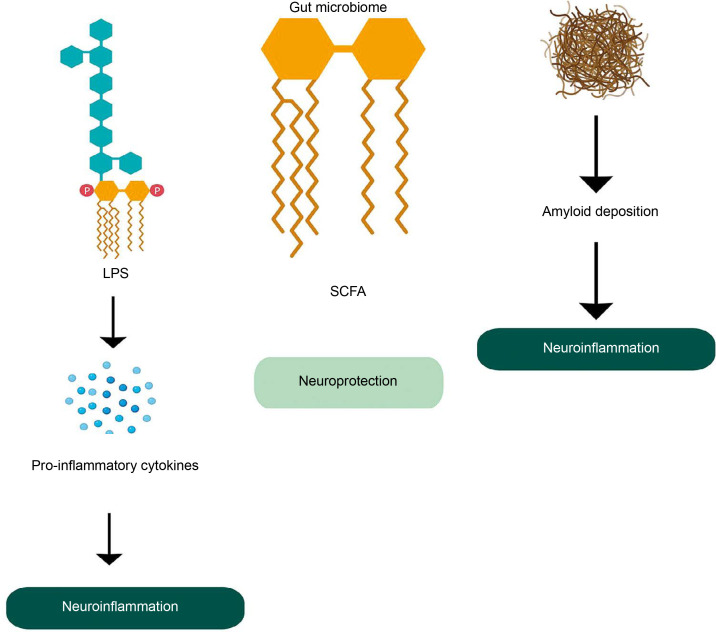
Role of gut microbiome in Alzheimer’s disease

The gut microbiota plays a critical role in maintaining brain health through the microbiome-gut-brain axis, facilitating bidirectional communication. Gut microbes produce a variety of metabolites that influence brain function and AD development. Dysregulated gut microbiota is associated with systemic inflammation, which can impair the blood–brain barrier (BBB) and promote neuroinflammation (Quigley, 2017). Dysregulation of tryptophan metabolism, regulated by gut microbes, has also been linked to the development of AD and other neurological disorders (Ruddick et al., 2006). Metabolites such as SCAFs, including butyrate, acetate, and propionate, have neuroprotective effects and can even repair a damaged BBB. However, the accumulation of microbiota-derived amyloids (certain lipoproteins that can form deposits of insoluble, β-plated structures e.g., curli fiber synthesized by *E.coli*) can be associated with inflammation at the site of amyloid deposition and upregulation of a chronic inflammatory network that is an important driving force in neuroinflammation in AD (Figure 4) (Zhao and Lukiw, 2015; Sochocka et al., 2019). Also, lipopolysaccharides produced by certain bacteria can induce proinflammatory pathways which cause pro-inflammatory cytokines to penetrate the BBB and produce reactive oxygen species. This is directly related to neuroinflammation and neurodegeneration in the brain (Zhang et al., 2009).

A study conducted at the Wisconsin Alzheimer’s Disease Research Center in the USA revealed significant differences in gut microbiota composition between AD patients and healthy individuals (Vogt et al., 2017). In AD patients, the abundance of *Firmicutes* and *Actinobacteria* is decreased, while *Proteobacteria* are proportionally increased.

A high-fat diet has been shown to promote the deposition of αβ plaques in the brain, as observed in APP/PS1, APP23, and APPNL-F models of amyloidosis. In contrast, consuming fish oil rich in omega-3 fatty acids enhances beneficial gut microbes such as *Bifidobacterium*, *Lactobacillus*, and *Akkermansia muciniphila*, which can reduce inflammation. Polyphenol-rich foods like blueberries, grape seed extract, green tea, pomegranates, coffee, and red wine increase the abundance of *Bifidobacterium* and *Lactobacillus*, further contributing to reduced inflammation.

Dietary fiber from plant-based foods also positively affects the gut microbiota, increasing levels of *Bifidobacterium*, *Lactobacillus*, and *Roseburia*. These bacteria metabolize complex carbohydrates into SCFAs, which are transported to the brain and exert neuroprotective effects (Zhu et al., 2023). Red wine consumption has been associated with preventing the generation of αβ peptides and reducing AD pathology.

Disrupted sleep or circadian rhythms can lead to gut microbiota dysbiosis, which may trigger neuroinflammation and contribute to AD pathology, though establishing a direct correlation remains challenging. Exercise has been shown to enhance the production of neuroprotective SCFAs, but its direct link to AD prevention is also not fully understood (Chandra, Sisodia and Vassar, 2023).

#### Parkinson’s disease

PD is a slowly progressive neurodegenerative disorder characterized by motor and non-motor symptoms. It involves a continuous loss of dopaminergic and cholinergic neurons along with the accumulation and aggregation of α-synuclein in the CNS. Although lifestyle factors have been studied in relation to PD, the lack of definitive data and conflicting results make clinical recommendations challenging. Increasing evidence points to an association between PD and the gut microbiome, often referred to as the “second brain” (Poewe et al., 2017; Ilie et al., 2020). A connection between enteric neurons and gut microbiota has been established, showing modulation of the hypothalamic–pituitary–adrenal (HPA) axis. Imbalances in gut microbiota are linked to low-grade inflammation, cellular degeneration, oxidative stress, and compromised BBB integrity (Stolp et al., 2005; Noble, Hsu and Kanoski, 2017).

The gut microbiome also affects enteric neurons responsible for α-synuclein production. For instance, *Enterococcus faecalis* and *Eggerthella lenta*, part of the gut microbiota, can metabolize Levodopa (L-Dopa), the primary drug used in PD treatment, interfering with its ability to cross the BBB. Infections with microbes such as *Helicobacter pylori* have been shown to influence PD by regulating dopamine levels in the brain. However, no conclusive inference can be drawn regarding the effect of *Helicobacter pylori* infection on PD due to the lack of clinical trials (Ilie et al., 2020). Also, another report demonstrated that the predisposition of PD is higher in persons with previous records of gastrointestinal problems. Unbalanced ROS generation has also been correlated with PD (Ilie et al., 2020). The causal relationship between dysbiotic microbiota and PD development remains uncertain. It is debated whether diet-induced changes in gut microbiota trigger PD or whether PD itself drives microbiota alterations. High milk consumption has been linked to PD development, and an intriguing study identified *Lactococcus* bacteriophages as negative modulators of the gut microbiome in PD (Tetz et al., 2018). Recent research highlights the role of circadian rhythm regulators, such as Bmal1, in influencing gut microbiota abundance. Bmal1-mediated regulation of immune genes, oscillatory production of immunoglobulin A, and ROS generation have been linked to both PD and AD (Khezri, Esmaeili and Ghasemnejad-Berenji, 2023).

#### Multiple sclerosis

Multiple sclerosis (MS) is a chronic autoimmune disorder characterized by demyelination of the CNS, leading to progressive and irreversible neurological disability. The disease is influenced by both genetic predisposition and environmental or lifestyle factors, including tobacco smoking, obesity during adolescence, lack of sun exposure, and circadian rhythm disruptions caused by night shift work (Alfredsson and Olsson, 2019). Experimental autoimmune encephalomyelitis, an animal model of MS, has demonstrated the involvement of intestinal microorganisms in disease pathology. Gut microbes induce pro-inflammatory responses through Th17 cells, exacerbating the condition. The diet also plays a significant role in MS pathogenesis. A multicenter study involving pediatric MS patients showed that increased saturated fat intake correlated with disease relapse, while adherence to a Mediterranean diet was associated with greater microbial diversity and the production of beneficial microbial metabolites. These dietary factors improved physical and cognitive symptoms, though they did not alleviate disease-related disabilities (Bohlouli et al., 2022).

#### Depression and anxiety

Lifestyle factors such as poor diet, lack of exercise, and smoking significantly contribute to the progression of psychiatric disorders like depression and anxiety. Consuming dietary elements like zinc, magnesium, vitamin B, and healthy fats such as olive oil is associated with a reduced risk of depression. Conversely, a high intake of red and processed meats, desserts, and fried foods promotes systemic inflammation, increasing the likelihood of depression and anxiety (Quirk et al., 2013).

Depression is characterized by low-grade inflammatory responses and activation of cell-mediated immunity. It triggers the anti-inflammatory reflex system and oxidative and nitrosative stress, which can lead to autoimmune responses against these stresses.

The human gut microbiome interacts with the CNS through a communication pathway named as microbiotagut-brain axis and it influences the pathophysiology of CNS diseases (Peirce and Alviña, 2019). The disruption of the microbiota-gut-brain axis also results in the dysregulation of the hypothalamic–pituitary–adrenal (HPA) axis which plays a crucial role in the development of depression. Studies reveal that depression is associated with reduced microbial diversity and species richness, alongside elevated levels of IL-6, IL-8, TNF-α, and C-reactive protein. Specific bacteria, such as *Eggerthella* (Kelly et al., 2016; Valles-Colomer et al., 2019), *Sellimonas*, *Lachnoclostridium*, and *Hungatella* show strong correlations with the severity of depressive and anxiety disorders. A healthy diet can positively influence the gut microbiota and strengthen the gut epithelial barrier, inhibiting depression progression. Antidepressant dietary options include foods rich in ω-3 polyunsaturated fatty acids (PUFAs) like fish oil (particularly docosahexaenoic acid), as well as fruits, vegetables, and polyphenol-rich foods, which modulate the gut microbiome and support mental health.

### Systemic inflammatory arthritis

Studies in animal models have demonstrated that gut microbiota plays a significant role in the development of systemic inflammatory diseases like rheumatoid arthritis. Dysbiotic gut microbiota can activate inflammatory pathways, particularly through toll-like receptor signaling, contributing to disease pathology. Lifestyle factors, especially diet, are pivotal in managing these diseases. Strong evidence from multiple randomized controlled trials indicates that a Mediterranean diet – rich in fruits, vegetables, whole grains, fish, and olive oil, with minimal red meat – has a positive effect on rheumatoid arthritis patients (Clemente, Manasson and Scher, 2018). Both vegetarian and Mediterranean diets enhance the production of SCFAs, which suppress undesirable inflammatory reactions.

## Effect of COVID-19 on the development of lifestyle diseases

COVID-19 has been associated with significant alterations in gut microbiota, characterized by an enrichment of opportunistic pathogens and a reduction in beneficial commensals. These changes persist even a year after recovery and may trigger IBS. Bacteria such as *Actinomyces oris, Clostridium* spp. (*C. clostridioforme*, *C. innocuum*), *Eggerthella lenta*, *F. plautii*, *Gordonibacter pamelaeae*, *Hungatella hathewayi*, *Rothia dentocariosa*, *Streptococcus* spp. (*S. anginosus* group, *S. gordonii*, *S. mitis*, *S. oralis*, *S. sanguinis*) are elevated in both COVID-19 and IBS. *E. lenta* can also be associated with cancer, diabetes, and CVDs. Long COVID has also altered the trajectory of many chronic diseases, particularly cardiovascular conditions such as myocardial inflammation, myocardial infarction, and right ventricular dysfunction (Raman et al., 2022). SARS-CoV-2 can cross the BBB, causing neuroinflammation associated with symptoms like brain fog, memory issues, attention disorders, sleep disturbances, anxiety, and depression. Additionally, COVID-19-induced microbial dysbiosis and disrupted intestinal barrier function can worsen pulmonary diseases, neurological disorders, and liver inflammation via the translocation of pathological bacteria and metabolites through the portal vein (Naidu et al., 2024).

## Microbial intervention in various diseases

**Figure 5:**
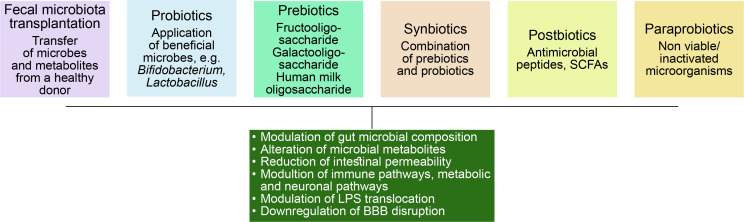
Gut microbiome modulation by various methods

Gut microbiome modulation can be an interesting solution to the problem of these lifestyle diseases. Figure 5 demonstrates different approaches taken in this regard.

### Fecal microbiota transplantation

It is characterized by the transfer of stool samples from healthy donors along with microbes and their metabolites to recipients. This method is currently being used to treat Clostridium difficile infection, along with antibiotics.

### Use of probiotics

Probiotics are the use of live microbes to treat gut microbiome dysbiosis and to bring back intestinal microflora balance. Probiotics are mostly comprised of *Bifidobacterium* and *Lactobacillus.* The metabolites derived from these microbes play a crucial role in host–microbe interaction and host health modulation by activating the immune system, improving intestinal barrier function and exerting antioxidant, anti-inflammatory effects, and possibly modulating gut microbiome composition and metabolome.

### Use of prebiotics

These compounds are nondigestible oligosaccharides (NDO), human milk oligosaccharides (HMOs), and soluble, fermentable fibers. Although these are known to regulate gut microbial flora, only a small number of studies are available showing the benefit. A study with galacto and fructo-oligosaccharide on male mice showed that these compounds have antidepressant properties.

### Use of synbiotics

In synbiotic therapy, both prebiotics and probiotics are used together where prebiotics favor the growth, viability, and metabolism of the probiotic microbes. This approach was found to be a success in decreasing the symptoms of depression.

### Use of postbiotics

Postbiotics/metabiotics/biogenics or cell-free supernatants consist of bacterial cell lysis products such as SCFAs, antimicrobial peptides, enzymes, teichoic acids, etc.

### Use of para probiotics

These are nonviable or inactivated microbial cells and may trigger the biological activity of the host if incorporated in a proper amount.(Sorboni et al., 2022).

## Conclusions

The relationship of gut microbiota and its host is a multifactorial complex interactome and is unique for its human host. There is growing attention to the characterization of gastrointestinal microbiota and its functionalities. Lifestyle diseases are non-communicable diseases like cardiovascular problems, cancer, etc which cause significant mortality and morbidity worldwide. Intestinal microbiota can be implicated in the pathogenesis of these diseases and demonstrates a complex relationship between the environmental factors, bacteria, derived metabolites, and the host that is characterized by complicated gene-environment interaction. Altered microbial metabolites due to microbial dysbiosis play crucial roles in the genesis and prognosis of these diseases. Therefore, manipulation of the gut microbiome in line with the personalized requirement can be a futuristic model of treatment for these diseases.

## Future direction

With the recent technologies, the knowledge about gut microbiome is continuously expanding. However, in addition to the bacterial research, other nonbacterial microbes of the human gut need to be studied, including bacteriophages, other viruses, eukaryotes, yeasts, and archaea. How do different factors modify the gut microbiome from the time of birth and how do these microbial modifications influence health, may provide new insight into the treatment of many challenging diseases like fibromyalgia, autism, different cancers, etc. It is of utmost importance to decipher the intricate relationship between gut microbiome and non-communicable lifestyle diseases, especially the identification of keystone species that are etiological or therapeutic towards a particular disease is immensely important. There is a lack of studies establishing the causality of a particular species from the gut and a particular lifestyle disease. There are many strong correlations observed in pre-clinical studies in animal models, that cannot be replicated in humans, impairing the translation and extrapolation of the findings to human scenarios. Moreover, the huge variation in individual gut microbiome composition further complicates the situation. Most human studies are on compositional variation of gut microbiota in different diseases while a deeper understanding of microbial mechanisms is more useful. Lastly, selecting a particular actionable microbe to be used in the treatment of diseases from a large interdependent consortium of gut microbiota is a daunting task. However, computational prediction models enabled by artificial intelligence and machine learning hold great promise in the field of clinical management. Along with this, dietary intervention, and the application of probiotics/prebiotics can help treat these diseases. In these regards, microbiome checking at different life stages are required for the intervention procedures to get optimized.

## References

[cit0001] Ahmad A.F., Dwivedi G., Fergal O’G., Caparros-Martin J., Ward N.C. (2019) The gut microbiome and cardiovascular disease: current knowledge and clinical potential. Am. J. Physiol. Heart Circ. Physiol. 317(5): H923–H938. 10.1152/ajpheart.00376.2019. 31469291

[cit0002] Alfredsson L., Olsson T. (2019) Lifestyle and environmental factors in multiple sclerosis. Cold Spring Harb. Perspect. Med. 9(4): a028944. 10.1101/cshperspect.a028944. 29735578 PMC6444694

[cit0003] Altman B.J. (2016) Cancer clocks out for lunch: disruption of circadian rhythm and metabolic oscillation in cancer. Front. Cell Dev. Biol. 4: 62. 10.3389/fcell.2016.00062. 27500134 PMC4971383

[cit0004] Alzheimer’s Association, Thies W., Bleiler L. (2013) 2013 Alzheimer’s disease facts and figures. Alzheimer’s Dement. 9(2): 208–245. 10.1016/j.jalz.2013.02.003. 23507120

[cit0005] An D., Oh S.F., Olszak T., Neves J.F., Avci F.Y., Erturk-Hasdemir D., Lu X., Zeissig S., Blumberg R.S., Kasper D.L. (2014) Sphingolipids from a symbiotic microbe regulate homeostasis of host intestinal natural killer T cells. Cell 156 (1–2): 123–133. 10.1016/j.cell.2013.11.042. 24439373 PMC3909465

[cit0006] Askarova S., Umbayev B., Masoud A-R., Kaiyrlykyzy A., Safarova Y., Tsoy A., Olzhayev F., Kushugulova A. (2020) The links between the gut microbiome, aging, modern lifestyle and Alzheimer’s disease. Front. Cell. Infect. Microbiol. 10: 104. 10.3389/fcimb.2020.00104. 32257964 PMC7093326

[cit0007] Baba Y., Iwatsuki M., Yoshida N., Masayuki W., Hideo Babaet H. (2017) Review of the gut microbiome and esophageal cancer: pathogenesis and potential clinical implications. Ann. Gastroenterol. Surg. 1(2): 99–104. 10.1002/ags3.12014. 29863142 PMC5881342

[cit0008] Barcik W., Boutin R.C.T., Sokolowska M., Finlayet B. (2020) The role of lung and gut microbiota in the pathology of asthma. Immunity 52(2): 241–255. 10.1016/j.immuni.2020.01.007. 32075727 PMC7128389

[cit0009] Beilfuss J., Berg V., Sneve M., Jorde R., Kamychevaet E. (2012) Effects of a 1-year supplementation with cholecalciferol on interleukin-6, tumor necrosis factor-alpha and insulin resistance in overweight and obese subjects. Cytokine 60(3): 870–874. 10.1016/j.cyto.2012.07.032. 22925537

[cit0010] Berk M., Williams L.J., Jacka F.N., O’Neil A., Pasco J.A., Moylan S., Allen N.B., Stuart A.L., Hayley A.C., Byrne M.L., Maes M. (2013) So depression is an inflammatory disease, but where does the inflammation come from? BMC Med. 11: 200. 10.1186/1741-7015-11-200. 24228900 PMC3846682

[cit0011] Bohlouli J., Namjoo I., Borzoo-Isfahani M., Poorbaferani F., Moravejolahkami A.R., Clark C. C T, Kermani M.A.H (2022) Modified Mediterranean diet v. traditional Iranian diet: efficacy of dietary interventions on dietary inflammatory index score, fatigue severity and disability in multiple sclerosis patients. Br. J. Nutr. 128(7): 1274–1284. 10.1017/S000711452100307X. 34392854

[cit0012] Bowerman KL, Rehman SF, Vaughan A, Lachner N, Budden KF, Kim RY, Wood DLA, Gellatly SL, Shukla SD, Wood LG, et al. (2020) Disease-associated gut microbiome and metabolome changes in patients with chronic obstructive pulmonary disease. Nat. Commun. 11(1): 5886. 10.1038/s41467-020-19701-0. 33208745 PMC7676259

[cit0013] Bu J., Wang Z. (2018) Cross-talk between gut microbiota and heart via the routes of metabolite and immunity. Gastroenterol. Res. Pract. 2018: 6458094. 10.1155/2018/6458094. 29967639 PMC6008745

[cit0014] Caramori G., Ruggeri P., Mumby S., Ieni A., Lo Bello F., Chimankar V., Donovan C., Andò F., Nucera F., Coppolino I., et al. (2019) Molecular links between COPD and lung cancer: new targets for drug discovery? Expert Opin. Ther. Targets 23(6): 539–553. 10.1080/14728222.2019.1615884. 31079559

[cit0015] Chandra S., Sisodia S.S., Vassar R.J. (2023) The gut microbiome in Alzheimer’s disease: what we know and what remains to be explored. Mol. Neurodegener. 18(1): 9. 10.1186/s13024-023-00595-7. 36721148 PMC9889249

[cit0016] Chen J., Liu X., Zou Y., Gong J., Ge Z., Lin X., Zhang W., Huang H., Zhao J., Saw P.E., et al. (2024) A high-fat diet promotes cancer progression by inducing gut microbiotamediated leucine production and PMN-MDSC differentiation. Proc. Natl. Acad. Sci. U.S.A. 121(20): e2306776121. 10.1073/pnas.2306776121. 38709933 PMC11098111

[cit0017] Chen L., Liu B., Ren L., Du H., Fei C., Qian C., Li B., Zhang R., Liu H., Li Z. (2023) High-fiber diet ameliorates gut microbiota, serum metabolism, and emotional mood in type 2 diabetes patients. Front. Cell. Infect. Microbiol. 13: 1069954. 10.3389/fcimb.2023.1069954. 36794003 PMC9922700

[cit0018] Choi Y.J., Myung S.K., Lee J.H. (2018) Light alcohol drinking and risk of cancer: a meta-analysis of cohort studies. Cancer Res. Treat. 50(2): 474–487. 10.4143/crt.2017.094. 28546524 PMC5912140

[cit0019] Clemente J.C., Manasson J., Scher J.U. (2018) The role of the gut microbiome in systemic inflammatory disease. BMJ 360: j5145. 10.1136/bmj.j5145. 29311119 PMC6889978

[cit0020] Cohen A.J., Pope C.A. (1995) Lung cancer and air pollution. Environ. Health Perspect. 103 (Suppl. 8): 219–224. 10.1289/ehp.95103s8219. 8741787 PMC1518961

[cit0021] De Filippo C., Cavalieri D., Di Paola M., Ramazzotti M., Poullet J.B., Massart S., Collini S., Pieraccini G., Lionettiet P. (2010) Impact of diet in shaping gut microbiota revealed by a comparative study in children from Europe and rural Africa. Proc. Natl. Acad. Sci. U.S.A. 107(33): 14691–14696. 10.1073/pnas.1005963107. 20679230 PMC2930426

[cit0022] De Palma G. Nadal I., Medina M., Donat E., Ribes-Koninckx C., Calabuig M., Sanz Y., et al. (2010) Intestinal dysbiosis and reduced immunoglobulin-coated bacteria associated with celiac disease in children. BMC Microbiol. 10: 63. 10.1186/1471-2180-10-63. 20181275 PMC2843610

[cit0023] Di Gangi A., Di Cicco M.E., Comberiati P., Peroni D.G. (2020) Go with your gut: the shaping of T-cell response by gut microbiota in allergic asthma. Front. Immunol. 11: 1485. 10.3389/fimmu.2020.01485. 32760404 PMC7372123

[cit0024] Dieterich W., Schink M., Zopf Y. (2018) Microbiota in the gastrointestinal tract. Med. Sci. 6(4): 116. 10.3390/medsci6040116. PMC6313343 30558253

[cit0025] Donia M.S., Fischbach M.A. (2015) Small molecules from the human microbiota. Science 349(6246): 1254766. 10.1126/science.1254766. 26206939 PMC4641445

[cit0026] Durack J., Lynch S.V. (2019) The gut microbiome: relationships with disease and opportunities for therapy. J. Exp. Med. 216(1): 20–40. 10.1084/jem.20180448. 30322864 PMC6314516

[cit0027] Ellulu M.S. (2018) Obesity, hypertension, and type-2 diabetes mellitus: the interrelationships and the determinants among adults in Gaza City, Palestine. Osong Public Health Res. Perspect. 9(6): 289–298. 10.24171/j.phrp.2018.9.6.02. 30584492 PMC6296808

[cit0028] El-Sherif A. El-Sherif S., Taylor A.H., Ayakannu T. (2021) Ovarian cancer: lifestyle, diet and nutrition. Nutr. Cancer 73(7): 1092–1107. 10.1080/01635581.2020.1792948. 32674720

[cit0029] Emoto T., Yamashita T., Kobayashi T., Sasaki N., Hirota Y., Hayashi T., So A., Kasahara K., Yodoi K., Matsumoto T., et al. (2017) Characterization of gut microbiota profiles in coronary artery disease patients using data mining analysis of terminal restriction fragment length polymorphism: gut microbiota could be a diagnostic marker of coronary artery disease. Heart Vessels 32(1): 39–46. 10.1007/s00380-016-0841-y. 27125213

[cit0030] Gumz M.L., Shimbo D., Abdalla M., Balijepalli R.C, Benedict C., Chen Y., Earnest D.J., Gamble K.L., Garrison S.R., Gong M.C., et al. (2023) Toward precision medicine: circadian rhythm of blood pressure and chronotherapy for hypertension – 2021 NHLBI workshop report. Hypertension 80(3): 503–522. 10.1161/HYPERTENSIONAHA.122.19372. 36448463 PMC9931676

[cit0031] Gupta A., Osadchiy V., Mayer E.A. (2020) Brain-gut-microbiome interactions in obesity and food addiction. Nat. Rev. Gastroenterol. Hepatol. 17(11): 655–672. 10.1038/s41575-020-0341-5. 32855515 PMC7841622

[cit0032] Haslam D., Sattar N., Lean M. (2006) Obesity — time to wake up. BMJ 333(7569): 640–642. 10.1136/bmj.333.7569.640. 16990325 PMC1570821

[cit0033] Ilie O.D., Ciobica A., McKenna J., Doroftei B., Ioannis Mavroudis I. (2020) Minireview on the relations between gut microflora and Parkinson’s disease: further biochemical (oxidative stress), inflammatory, and neurological particularities. Oxid. Med. Cell. Longev. 2020: 4518023. 10.1155/2020/4518023. 32089768 PMC7025076

[cit0034] Janeiro M.H., Ramírez M.J., Milagro F.I., Martínez J.A., Solaset M. (2018) Implication of trimethylamine N-oxide (TMAO) in disease: potential biomarker or new therapeutic target. Nutrients 10(10): 1398. 10.3390/nu10101398. 30275434 PMC6213249

[cit0035] Kamiya T., Tang C., Kadoki M., Oshima K., Hattori M., Saijo S., Adachi Y., Ohno N., Iwakura Y., et al. (2018) β-glucans in food modify colonic microflora by inducing antimicrobial protein, calprotectin, in a dectin-1-induced-IL-17F-dependent manner. Mucosal Immunol. 11(3): 763–773. 10.1038/mi.2017.86. 29068000

[cit0036] Kasai C., Sugimoto K., Moritani I., Tanaka J., Oya Y., Inoue H., Tameda M., Shiraki K., Ito M., Takei Y., et al. (2015) Comparison of the gut microbiota composition between obese and non-obese individuals in a Japanese population, as analyzed by terminal restriction fragment length polymorphism and next-generation sequencing. BMC Gastroenterol. 15: 100. 10.1186/s12876-015-0330-2. 26261039 PMC4531509

[cit0037] Katzke V.A., Kaaks R., Kühn T. (2015) Lifestyle and cancer risk. Cancer J. 21(2): 104–110. 10.1097/PPO.0000000000000101. 25815850

[cit0038] Kelly J.R., Borre Y., O’ Brien C., Patterson E., Aidy S.E., Deane J., Kennedy P.J, Beers S., Scott K., Moloney G., et al. (2016) Transferring the blues: depression-associated gut microbiota induces neurobehavioural changes in the rat. J. Psychiatr. Res. 82: 109–118. 10.1016/j.jpsychires.2016.07.019. 27491067

[cit0039] Khan I., Ullah N., Zha L., Bai Y. Khan A., Zhao T., Che T., Chunjiang Zhang C. (2019) Alteration of gut microbiota in inflammatory bowel disease (IBD): cause or consequence? IBD treatment targeting the gut microbiome. Pathogens 8(3): 126. 10.3390/pathogens8030126. 31412603 PMC6789542

[cit0040] Khezri M.R., Esmaeili A., Ghasemnejad-Berenji M. (2023) Role of Bmal1 and gut microbiota in Alzheimer’s disease and Parkinson’s disease pathophysiology: the probable effect of melatonin on their association. ACS Chem. Neurosci. 14(21): 3883–3893. 10.1021/acschemneuro.3c00418. 37823531

[cit0041] Kilkkinen A., Rissanen H., Klaukka T., Pukkala E., Heliövaara M., Huovinen P., Männistö S., Aromaa A., Knekt P. (2008) Antibiotic use predicts an increased risk of cancer. Int. J. Cancer 123(9): 2152–2155. 10.1002/ijc.23622. 18704945

[cit0042] Kim J.H., Oh C.M., Yoo J.H. (2023) Obesity and novel management of inflammatory bowel disease. World J. Gastroenterol. 29(12): 1779–1794. 10.3748/wjg.v29.i12.1779. 37032724 PMC10080699

[cit0043] Krautkramer K.A., Kreznar J.H, Romano K.A, Vivas E.I, Barrett-Wilt G.A, Rabaglia M.E, Keller M.P, Attie A.D, Rey F.E, Denu J.M. (2016) Diet-microbiota interactions mediate global epigenetic programming in multiple host tissues. Mol. Cell 64(5): 982–992. 10.1016/j.molcel.2016.10.025. 27889451 PMC5227652

[cit0044] Kronman M.P., Zaoutis T.E., Haynes K., Feng R., Coffin S.E. (2012) Antibiotic exposure and IBD development among children: a population-based cohort study. Pediatrics 130(4): e794–e803. 10.1542/peds.2011-3886. 23008454 PMC4074626

[cit0045] Kwon H., Pessin J.E. (2013) Adipokines mediate inflammation and insulin resistance. Front. Endocrinol. 4: 71. 10.3389/fendo.2013.00071. PMC3679475 23781214

[cit0046] Larsen N., Vogensen F.K., van den Berg F., Nielsen D.S., Andreasen A.S., Pedersen B.K., Al-Soud W.A., Sørensen S.J., Hansen L.H., Jakobsen M. (2010) Gut microbiota in human adults with type 2 diabetes differs from non-diabetic adults. PLoS ONE 5(2): e9085. 10.1371/journal.pone.0009085. 20140211 PMC2816710

[cit0047] Lavelle A., Sokol H. (2020) Gut microbiota-derived metabolites as key actors in inflammatory bowel disease. Nat. Rev. Gastroenterol. Hepatol. 17(4): 223–237. 10.1038/s41575-019-0258-z. 32076145

[cit0048] Lee C.J., Sears C.L., Maruthur N. (2020) Gut microbiome and its role in obesity and insulin resistance. Ann. N. Y. Acad. Sci. 1461(1): 37–52. 10.1111/nyas.14107. 31087391

[cit0049] Lee M., Chang E.B. (2021) Inflammatory bowel diseases (IBD) and the microbiome—searching the crime scene for clues. Gastroenterology 160(2): 524–537. 10.1053/j.gastro.2020.09.056. 33253681 PMC8098834

[cit0050] Lee W.J., Lim J.E., Kang J.O., Ha T.W., Jung H.U., Kim D.J., Baek E.J., Kim H.K., Chung J.Y., Oh B., et al. (2022) Smoking-interaction loci affect obesity traits: a gene-smoking stratified meta-analysis of 545,131 Europeans. Lifestyle Genom. 15(3): 87–97. 10.1159/000525749. 35793639

[cit0051] Levan S.R., Stamnes K.A., Lin D.L., Panzer A.R., Fukui E., McCauley K., Fujimura K.E., McKean M., Ownby D.R., Zoratti E.M., et al. (2019) Elevated faecal 12,13-diHOME concentration in neonates at high risk for asthma is produced by gut bacteria and impedes immune tolerance. Nat. Microbiol. 4(11): 1851–1861. 10.1038/s41564-019-0498-2. 31332384 PMC6830510

[cit0052] Lewis J.D., Chen E.Z., Baldassano R.N., Otley A.R., Griffiths A.M., Lee D., Bittinger K., Bailey A., Elliot S., Friedman E.S., et al. (2015) Inflammation, antibiotics, and diet as environmental stressors of the gut microbiome in pediatric Crohn’s disease. Cell Host Microbe 18(4): 489–500. 10.1016/j.chom.2015.09.008. 26468751 PMC4633303

[cit0053] Li N., Dai Z., Wang Z., Deng Z., Zhang J., Pu J., Cao W., Pan T., Zhou Y., Yang Z., et al. (2021) Gut microbiota dysbiosis contributes to the development of chronic obstructive pulmonary disease. Respir. Res. 22(1): 274. 10.1186/s12931-021-01872-z. 34696775 PMC8543848

[cit0054] Li Q., Jin M., Liu Y., Jin L. (2020) Gut microbiota: its potential roles in pancreatic cancer. Front. Cell. Infect. Microbiol. 10: 572492. 10.3389/fcimb.2020.572492. 33117731 PMC7575684

[cit0055] Liang L., Ai L., Qian J., Fang J.Y., Xu J. (2015) Long noncoding RNA expression profiles in gut tissues constitute molecular signatures that reflect the types of microbes. Sci. Rep. 5: 11763. 10.1038/srep11763. 26123364 PMC4485256

[cit0056] Liu J.L., Xu X., Rixiati Y., Wang C.Y, Ni H.L, Chen W.S, Gong H.M., Zhang Z.L., Li S., Shen T., et al. (2024) Dysfunctional circadian clock accelerates cancer metastasis by intestinal microbiota triggering accumulation of myeloidderived suppressor cells. Cell Metab. 36(6): 1320–1334.e9. 10.1016/j.cmet.2024.04.019. 38838643

[cit0057] Maki K.A., Burke L.A., Calik M.W., Watanabe-Chailland M., Sweeney D., Romick-Rosendale L.E., Green S.J., Fink A.M. (2020) Sleep fragmentation increases blood pressure and is associated with alterations in the gut microbiome and fecal metabolome in rats. Physiol. Genomics 52(7): 280–292. 10.1152/physiolgenomics.00039.2020. 32567509 PMC7468692

[cit0058] Manrique P., Bolduc B., Walk S.T, van der Oost J., de Vos W.M., Young M.J. (2016) Healthy human gut phageome. Proc. Natl. Acad. Sci. U.S.A. 113(37): 10400–10405. 10.1073/pnas.1601060113. 27573828 PMC5027468

[cit0059] Matsushita M., Fujita K., Hayashi T., Kayama H., Motooka D., Hase H., Jingushi K., Yamamichi G., Yumiba S., Tomiyama E., et al. (2021) Gut microbiota-derived short-chain fatty acids promote prostate cancer growth via IGF1 signaling. Cancer Res. 81(15): 4014–4026. 10.1158/0008-5472.CAN-20-4090. 34039634

[cit0060] Münch N.S., Fang H.Y., Ingermann J., Maurer H C., Anand A., Kellner V., Sahm V., Wiethaler M., Baumeister T., Wein F., et al. (2019) High-fat diet accelerates carcinogenesis in a mouse model of Barrett’s esophagus via interleukin 8 and alterations to the gut microbiome. Gastroenterology 157(2): 492–506.e2. 10.1053/j.gastro.2019.04.013. 30998992 PMC6662596

[cit0061] Naidu A.S., Wang C.K., Rao P., Mancini F., Clemens R.A., Wirakartakusumah A., Chiu H.F., Yen C.H., Porretta S., Mathai I., et al. (2024) Precision nutrition to reset virusinduced human metabolic reprogramming and dysregulation (HMRD) in long-COVID. NPJ Sci. Food 8(1): 19. 10.1038/s41538-024-00261-2. 38555403 PMC10981760

[cit0062] Nemet I., Saha P.P., Gupta N., Zhu W., Romano K.A., Skye S.M., Cajka T., Mohan M.L, Lin L., Wu Y., et al. (2020) A cardiovascular disease-linked gut microbial metabolite acts via adrenergic receptors. Cell 180(5): 862–877.e22. 10.1016/j.cell.2020.02.016. 32142679 PMC7402401

[cit0063] Noble E.E., Hsu T.M., Kanoski S.E. (2017) Gut-to-brain dysbiosis: mechanisms linking Western diet consumption, the microbiome, and cognitive impairment. Front. Behav. Neurosci. 11: 9. 10.3389/fnbeh.2017.00009. 28194099 PMC5277010

[cit0064] Oddy W.H. (2017) Breastfeeding, childhood asthma, and allergic disease. Ann. Nutr. Metab. 70(Suppl. 2): 26–36. 10.1159/000457920. 28521318

[cit0065] Oduaran O.H., Tamburini F.B., Sahibdeen V., Brewster R., Gómez-Olivé F.X., Kahn K., Norris S.A., Tollman S.M., Twine R., Wade A.N., et al. (2020) Gut microbiome profiling of a rural and urban South African cohort reveals biomarkers of a population in lifestyle transition. BMC Microbiol. 20(1): 330. 10.1186/s12866-020-02017-w. 33129264 PMC7603784

[cit0066] Ottiger M., Nickler M., Steuer C., Bernasconi L., Huber A., Christ-Crain M., Henzen C., Hoess C., Thomann R., Zimmerli W., et al. (2018) Gut, microbiota-dependent trimethylamine-N-oxide is associated with long-term all-cause mortality in patients with exacerbated chronic obstructive pulmonary disease. Nutrition. 45: 135–141.e1. 10.1016/j.nut.2017.07.001. 28870405

[cit0067] Pasini E., Aquilani R., Testa C., Baiardi P., Angioletti S., Boschi F., Verri M., Dioguardi F. (2016) Pathogenic gut flora in patients with chronic heart failure. JACC Heart Fail. 4(3): 220–227. 10.1016/j.jchf.2015.10.009. 26682791

[cit0068] Peirce J.M., Alviña K. (2019) The role of inflammation and the gut microbiome in depression and anxiety. J. Neurosci. Res. 97(10): 1223–1241. 10.1002/jnr.24476. 31144383

[cit0069] Poewe W., Seppi K., Tanner C.M, Halliday G.M., Brundin P., Volkmann J., Schrag A.E., Lang A.E. (2017) Parkinson disease. Nat. Rev. Dis. Primers 3(1): 17013. 10.1038/nrdp.2017.13. 28332488

[cit0070] Qin J., Li Y., Cai Z., Li S., Zhu J., Zhang F., Liang S., Zhang W., Guan Y., Shen D., et al. (2012) A metagenome-wide association study of gut microbiota in type 2 diabetes. Nature 490(7418): 55–60. 10.1038/nature11450. 23023125

[cit0071] Quigley E.M.M. (2017) Microbiota-brain-gut axis and neurodegenerative diseases. Curr. Neurol. Neurosci. Rep. 17(12): 94. 10.1007/s11910-017-0802-6. 29039142

[cit0072] Quirk S.E., Williams L.J., O’Neil A., Pasco J.A., Jacka F.N., Housden S., Berk M., Brennan S.L. (2013) The association between diet quality, dietary patterns and depression in adults: a systematic review. BMC Psychiatry 13: 175. 10.1186/1471-244X-13-175. 23802679 PMC3706241

[cit0073] Raman B., Bluemke D.A., Lüscher T.F., Neubauer S. (2022) Long COVID: post-acute sequelae of COVID-19 with a cardiovascular focus. Eur. Heart J. 43(11): 1157–1172. 10.1093/eurheartj/ehac031. 35176758 PMC8903393

[cit0074] Reitmeier S., Kiessling S., Clavel T., List M., Almeida E.L., Ghosh T.S., Neuhaus K., Grallert H., Linseisen J., Skurk T., et al. (2020) Arrhythmic gut microbiome signatures predict risk of type 2 diabetes. Cell Host Microbe 28(2): 258–272.e6. 10.1016/j.chom.2020.06.004. 32619440

[cit0075] Rieck G., Fiander A. (2006) The effect of lifestyle factors on gynecological cancer. Best Pract. Res. Clin. Obstet. Gynaecol. 20(2): 227–251. 10.1016/j.bpob-gyn.2005.10.010. 16543119

[cit0076] Rigotti N.A., Clair C. (2013) Managing tobacco use: the neglected cardiovascular disease risk factor. Eur. Heart J. 34(42): 3259–3267. 10.1093/eurheartj/eht352. 24014389

[cit0077] Rossi M., Amaretti A., Raimondi S. (2011) Folate production by probiotic bacteria. Nutrients 3(1): 118–134. 10.3390/nu3010118. 22254078 PMC3257725

[cit0078] Ruddick J.P., Evans A.K, Nutt D.J., Lightman S.L., Rook G.A.W., Lowry C.A. (2006) Tryptophan metabolism in the central nervous system: medical implications. Expert Rev. Mol. Med. 8(20): 1–27. 10.1017/S1462399406000068. 16942634

[cit0079] Segata N., Haake S.K., Mannon P., Lemon K.P., Waldron L., Gevers D., Huttenhower C., Izard J. (2012) Composition of the adult digestive tract bacterial microbiome based on seven mouth surfaces, tonsils, throat and stool samples. Genome Biol. 13(6): R42. 10.1186/gb-2012-13-6-r42. 22698087 PMC3446314

[cit0080] Shapiro H., Goldenberg K., Ratiner K., Elinav E (2022) Smoking-induced microbial dysbiosis in health and disease. Clin. Sci. 136(18): 1371–1387. 10.1042/CS20220175. PMC9527826 36156126

[cit0081] Shearer W.T., Reuben J.M., Mullington J.M., Price N.J., Lee B.N., Smith E.O., Szuba M.P., Van Dongen H.P., Dinges D.F. (2001) Soluble TNF-α receptor 1 and IL-6 plasma levels in humans subjected to the sleep deprivation model of spaceflight. J. Allergy Clin. Immunol. 107(1): 165–170. 10.1067/mai.2001.112270. 11150007

[cit0082] Shephard R.J., Freedson P. (1996) Exercise and cancer: linkages with obesity? Crit. Rev. Food Sci. Nutr. 36(4): 321–339. 10.1080/10408399609527728. 8740437

[cit0083] Shi N., Li N., Duan X., Niu H. (2017) Interaction between the gut microbiome and mucosal immune system. Mil. Med. Res. 4: 14. 10.1186/s40779-017-0122-9. 28465831 PMC5408367

[cit0084] Sikalidis A.K., Maykish A. (2020) The gut microbiome and type 2 diabetes mellitus: discussing a complex relationship. Biomedicines 8(1): 8. 10.3390/biomedicines8010008. 31936158 PMC7168169

[cit0085] Sochocka M., Donskow-Łysoniewska K., Diniz B.S., Kurpas D., Brzozowska E., Leszek J. (2019) The gut microbiome alterations and inflammation-driven pathogenesis of Alzheimer’s disease – a critical review. Mol. Neurobiol. 56(3): 1841–1851. 10.1007/s12035-018-1188-4. 29936690 PMC6394610

[cit0086] Song M., Chan A.T. (2019) Environmental factors, gut microbiota, and colorectal cancer prevention. Clin. Gastroenterol. Hepatol. 17(2): 275–289. 10.1016/j.cgh.2018.07.012. 30031175 PMC6314893

[cit0087] Sorboni S.G., Moghaddam H.S., Jafarzadeh-Esfehani R., Soleimanpour S. (2022) A comprehensive review on the role of the gut microbiome in human neurological disorders. Clin. Microbiol. Rev. 35(1): e0033820. 10.1128/CMR.00338-20. 34985325 PMC8729913

[cit0088] Stafstrom C.E., Rho J.M. (2012) The ketogenic diet as a treatment paradigm for diverse neurological disorders. Front. Pharmacol. 3: 59. 10.3389/fphar.2012.00059. 22509165 PMC3321471

[cit0089] Stolp H.B., Dziegielewska K.M., Ek C.J., Potter A.M., Saunders N.R., et al. (2005) Long-term changes in blood-brain barrier permeability and white matter following prolonged systemic inflammation in early development in the rat. Eur. J. Neurosci. 22(11): 2805–2816. 10.1111/j.1460-9568.2005.04483.x. 16324115

[cit0090] Takahashi K., Sugi Y., Hosono A., Kaminogawa S. (2009) Epigenetic regulation of TLR4 gene expression in intestinal epithelial cells for the maintenance of intestinal homeostasis. J. Immunol. 183(10): 6522–6529. 10.4049/jimmunol.0901271. 19846881

[cit0091] Tetz G., Brown S.M., Hao Y., Tetz V. (2018) Parkinson’s disease and bacteriophages as its overlooked contributors. Sci. Rep. 8(1): 10812. 10.1038/s41598-018-29173-4. 30018338 PMC6050259

[cit0092] Ticinesi A., Milani C., Lauretani F., Nouvenne A., Mancabelli L., Lugli G.A., Turroni F., Duranti S., Mangifesta M., Viappiani A., et al. (2017) Gut microbiota composition is associated with polypharmacy in elderly hospitalized patients. Sci. Rep. 7(1): 11102. 10.1038/s41598-017-10734-y. 28894183 PMC5593887

[cit0093] Tong Y., Gao H., Qi Q., Liu X., Li J., Gao J., Li P., Wang Y., Du L., Wang C., et al. (2021) High-fat diet, gut microbiome and gastrointestinal cancer. Theranostics 11(12): 5889–5910. 10.7150/thno.56157. 33897888 PMC8058730

[cit0094] Trompette A., Gollwitzer E.S., Pattaroni C., Lopez-Mejia I.C., Riva E., Pernot J., Ubags N., Fajas L., Nicod L.P., Marsland B.J. (2018) Dietary fiber confers protection against flu by shaping Ly6c – patrolling monocyte hematopoiesis and CD8+ T cell metabolism. Immunity 48(5): 992–1005.e8. 10.1016/j.immuni.2018.04.022. 29768180

[cit0095] Tsalamandris S., Antonopoulos A.S., Oikonomou E., Papamikroulis G.A., Vogiatzi G., Papaioannou S., Deftereos S., Tousoulis D. (2019) The role of inflammation in diabetes: current concepts and future perspectives. Eur. Cardiol. Rev. 14(1): 50–59. 10.15420/ecr.2018.33.1. PMC6523054 31131037

[cit0096] Tseng C.H., Wu C.Y. (2019) The gut microbiome in obesity. J. Formos. Med. Assoc. 118 (Suppl. 3): S3–S9. 10.1016/j.jfma.2018.07.009. 30057153

[cit0097] Valles-Colomer M., Falony G., Darzi Y., Tigchelaar E.F., Wang J., Tito R.Y., Schiweck C., Kurilshikov A., Joossens M., Wijmenga C., et al. (2019) The neuroactive potential of the human gut microbiota in quality of life and depression. Nat. Microbiol. 4(4): 623–632. 10.1038/s41564-018-0337-x. 30718848

[cit0098] Vaughan A., Frazer Z.A., Hansbro P.M., Yang I.A. (2019) COPD and the gut-lung axis: the therapeutic potential of fiber. J. Thorac. Dis. 11(Suppl. 17): S2173–S2180. 10.21037/jtd.2019.10.40. 31737344 PMC6831926

[cit0099] Vivarelli S., Salemi R., Candido S., Falzone L., Santagati M., Stefani S., Torino F., Banna G.L., Tonini G., Libra M. (2019) Gut microbiota and cancer: from pathogenesis to therapy. Cancers 11(1): 38. 10.3390/cancers11010038. 30609850 PMC6356461

[cit0100] Vogt N.M., Kerby R.L., Dill-McFarland K.A., Harding S.J., Merluzzi A.P., Johnson S.C., Carlsson C.M., Asthana S., Zetterberg H., Blennow K., et al. (2017) Gut microbiome alterations in Alzheimer’s disease. Sci. Rep. 7(1): 13537. 10.1038/s41598-017-13601-y. 29051531 PMC5648830

[cit0101] Wheeler M.L., Limon J.J., Bar A.S., Leal C.A., Gargus M., Tang J., Brown J., Funari V.A., Wang H.L., Crother T.R., et al. (2016) Immunological consequences of intestinal fungal dysbiosis. Cell Host Microbe 19(6): 865–873. 10.1016/j.chom.2016.05.003. 27237365 PMC4900921

[cit0102] Witkowski M., Weeks T.L., Hazen S.L. (2020) Gut microbiota and cardiovascular disease. Circ. Res. 127(4): 553–570. 10.1161/CIRCRESAHA.120.316242. 32762536 PMC7416843

[cit0103] Woo V., Alenghat T. (2022) Epigenetic regulation by gut microbiota. Gut Microbes 14(1): 2022407. 10.1080/19490976.2021.2022407. 35000562 PMC8744890

[cit0104] Wu H., Esteve E., Tremaroli V., Khan M.T., Caesar R., Mannerås-Holm L., Ståhlman M., Olsson L.M., Serino M., Planas-Fèlix M., et al. (2017) Metformin alters the gut microbiome of individuals with treatment-naive type 2 diabetes, contributing to the therapeutic effects of the drug. Nat. Med. 23(7): 850–858. 10.1038/nm.4345. 28530702

[cit0105] Xu H., Wang X., Feng W., Liu Q., Zhou S., Liu Q., Cai L. (2020) The gut microbiota and its interactions with cardiovascular disease. Microb. Biotechnol. 13(3): 637–656. 10.1111/1751-7915.13524. 31984651 PMC7111081

[cit0106] Zhang R., Miller R.G., Gascon R., Champion S., Katz J., Lancero M., Narvaez A., Honrada R., Ruvalcaba D., McGrath M.S. (2009) Circulating endotoxin and systemic immune activation in sporadic amyotrophic lateral sclerosis (sALS). J. Neuroimmunol. 206(1–2): 121–124. 10.1016/j.jneuroim.2008.09.017. 19013651 PMC2995297

[cit0107] Zhang X., Coker O.O., Chu E.S., Fu K., Lau H.C.H., Wang Y.X., Chan A.W.H., Wei H., Yang X., Joseph J.Y., et al. (2021) Dietary cholesterol drives fatty liver-associated liver cancer by modulating gut microbiota and metabolites. Gut 70(4): 761–774. 10.1136/gutjnl-2019-319664. 32694178 PMC7948195

[cit0108] Zhang Y.J., Li S., Gan R.Y., Zhou T., Xu D.P., Li H.B. (2015) Impacts of gut bacteria on human health and diseases. Int. J. Mol. Sci. 16(12): 7493–7519. 10.3390/ijms16047493. 25849657 PMC4425030

[cit0109] Zhao L. (2013) The gut microbiota and obesity: from correlation to causality. Nat. Rev. Microbiol. 11(9): 639–647. 10.1038/nrmicro3089. 23912213

[cit0110] Zhao Y., Lukiw W.J. (2015) Microbiome-generated amyloid and potential impact on amyloidogenesis in Alzheimer’s disease (AD). J. Nat. Sci. 1(7): e138. 26097896 PMC4469284

[cit0111] Zhu G., Zhao J., Zhang H., Wang G., Chen W. (2023) Gut microbiota and its metabolites: bridge of dietary nutrients and Alzheimer’s disease. Adv. Nutr. 14(4): 819–839. 10.1016/j.advnut.2023.04.005. 37075947 PMC10334159

